# Qualitative Exploration of Need for Adolescent Sexual and Reproductive Health Care and Service Pathways Readiness During Pandemic in Nepal

**DOI:** 10.1111/hex.70931

**Published:** 2026-10-16

**Authors:** Saki Thapa, Bikram Bucha, Swastika Shrestha, Raghu Dhital, Kritika Dixit, Lal Rawal, Amit Timilsina

**Affiliations:** ^1^ Research Department Birat Nepal Medical Trust (BNMT) Kathmandu Nepal; ^2^ Department of Global Public Health Karolinska Institutet Stockholm Sweden; ^3^ Public Health, School of Health Medical and Applied Science CQUniversity Sydney Australia; ^4^ Research and Community Development Center Kathmandu Nepal

**Keywords:** adolescents, COVID‐19, pandemics, reproductive rights, sexual and reproductive health, social cognitive theory

## Abstract

**Background:**

COVID‐19 pandemic disrupted adolescents' access to sexual and reproductive health (SRH) services for adolescents in Nepal. This study explored adolescents' and health care provider's perspectives on how the COVID‐19 pandemic affected adolescents' SRH information and services, SRH‐related behaviours, and factors that strengthen care and service pathways during the pandemic in Nepal.

**Methods:**

This study used qualitative methods and adapted the social cognitive theory as an analytical framework to explore individual, social and environmental influences on adolescents' sexual and reproductive health (SRH) information and services pathways during the COVID‐19 pandemic. A total of 86 participants were included in the study, including 64 adolescents (15–19 years) and 22 service providers. For this, total of six focus group discussions, 12 in‐depth interviews and 22 key informant interviews were conducted. Thematic analysis was conducted to identify, analyse and interpret the data.

**Result:**

The study findings showed that COVID‐19 disrupted adolescents' SRH information and services pathways through interconnected environmental, social and individual factors. School closures reduced access to school‐based SRH information and opportunities to interact with teachers and peers, while interruptions in service delivery compromised adolescent's pathways to SRH information and services. Adolescents reported changes in sexual behaviours and mental health effects during pandemic. Strengthening adolescents' pathways to SRH information and service during crises requires provider's capacity strengthening, conducting virtual child club programmes for self‐help and social interaction, digital information and psychosocial services, and prioritising coordinated interventions among stakeholders.

**Conclusion:**

Maintaining adolescent SRH care during public health emergencies requires an ecosystem approach addressing individual, social and environmental factors together. Adolescents' SRH needs during public health emergencies extend beyond the availability of clinical services and include opportunities for emotional, social and trusted support. Strengthening adolescents' self‐efficacy, conducive social environments, and community‐based actors such as Female Community Health Volunteers (FCHVs) along with accessible, confidential and adolescent‐responsive services, helps maintain continuity of sexual and reproductive health and rights (SRHR) information and care during emergencies.

**Patient or Public Contribution:**

This manuscript is developed from the study done under the larger project that was designed to address sexual and reproductive health and rights (SRHR) of adolescents and women in the Sindhupalchowk district. The study adopted a participatory approach through active engagement with adolescents, community actors, health service providers, and local stakeholders throughout the research process. Different consultation meetings were held with these stakeholders to identify research priorities, refining research questions and development of study tools to ensure contextual relevance and responsiveness to community SRHR needs. The findings further emphasise the importance of community‐based SRHR approaches, strengthening local actors and volunteers and fostering adolescent self‐efficacy in supporting adolescent responsive services during public health emergencies.

## 1 Introduction

On 11 March 2020, the World Health Organisation (WHO) declared the coronavirus disease (COVID‐19) outbreak as a global pandemic following its declaration as a global public health emergency on 30th January 2020 [1]. Governments across the world responded to contain the transmission and flatten the curve through stringent lockdowns, travel restrictions, closure of schools and workplaces and social distancing measures [2, 3, 4, 5]. A major impact of these measures and the pandemic was on access to sexual and reproductive health and rights (SRHR) by adolescents [6]. The Guttmacher Institute estimated that a decline of 12%–25% in essential sexual and reproductive health care in low‐ and middle‐income countries (LMICs), adolescents between the age group of 15–19 could experience an additional 743,000 unintended pregnancies, additional 134,000 major obstetric complications and an additional 3400 maternal deaths due to COVID‐19 induced disruptions [6]. Multiple studies conducted during the pandemic showed increased sexual activity and unprotected sex among adolescents and young people, a shift in contraceptive method and heightened service pathway gaps for marginalised young people as a consequence of the pandemic [7, 8, 9].

In Nepal, an LMIC in South Asia, access to and utilisation of SRH (sexual and reproductive health) services were already low prior to the pandemic. Adolescent girls marrying before the legal age of marriage in Nepal (20 years age) was 15.3%, 35% had unmet need for family planning, only 15% of married adolescent girls were using modern contraceptives and 17% teenage pregnancy in Nepal [10, 11]. The pandemic and lockdown further exacerbated the impact on SRH services in Nepal. A cross‐sectional study conducted in one tertiary centre in Nepal reported that 47% fewer women in Nepal visited safe abortion services during the lockdown than after lifting of the order [12]. In addition, institutional service utilisation declined markedly during the pandemic, which resulted in an increase in adverse outcomes such as stillbirths, which rose from 14 to 21 per 1000 total births during the early lockdown period [13]. However, studies about SRH information and service pathways particularly catered to adolescents are limited, including how the pandemic disrupted adolescents' SRH behaviour and their access to information and services in Nepal. Access to and utilisation of SRH information and services among adolescents involve an interconnected process through which adolescents obtain information, seek support, make decisions about SRH and reach and utilise appropriate services. These pathways are shaped by individual, social and health system factors that influence adolescents' access to information, support and services [14, 15]. Evidence from the COVID‐19 pandemic indicates that access to SRH information and services was disrupted by school closures, mobility restrictions and interruptions in health service delivery [8]. These disruptions may have affected the ways in which adolescents obtained information, sought support, made decisions about their SRH and accessed services. Understanding these processes during the pandemic can provide insights into the barriers and facilitators that influence adolescents' ability to obtain SRH information and utilise services during emergencies. Therefore, this study explored perspectives on (a) how the COVID‐19 pandemic affected access to and utilisation of SRH information and services among adolescents in Nepal and (b) identified barriers and facilitators influencing SRH information and service pathways among adolescents during the COVID‐19 pandemic in Nepal.

## 2 Methods and Materials

### 2.1 Study Design and Setting

An exploratory qualitative study was conducted to explore the impact of the COVID‐19 pandemic on adolescents' SRH access, behaviours and service utilisation in Nepal. The study used social cognitive theory [16], which emphasises the dynamic interaction of personal, behaviour and environmental influences to access and continue SRH service utilisation among adolescents in this study. This guided our data collection tools and interpretation of findings to explore how SRH behaviours of adolescents were shaped during the COVID‐19 pandemic.

The study was carried out in four municipalities of Sindhupalchowk district: Bharabise and Chautara municipalities; and Balephi and Indrawati rural municipalities. These municipalities were selected in consultation with the Health Office in Sindhupalchowk, and other concerned authorities such as health and education section of each municipality, ministry of health and population and adolescents from the SRHR project implemented by Birat Nepal Medical Trust (BNMT). Sindhupalchowk is predominantly rural with mountainous terrain and was severely affected by the 2015 earthquake [17]. This contributed to the persistent challenges in health service access and utilisation in the district [18, 19].

### 2.2 Study Participants and Sampling

The study participants comprised

a. adolescents
b. healthcare providers and other key stakeholders, including community‐based health actors, teachers, health system officials and
c. representatives from civil society organisations working on the SRH sector

The participants were purposively selected in consultation with health care providers, FCHVs and school teachers based on their knowledge and experience of SRH service utilisation (in case of adolescents) and delivery (in case of health providers). Purposive sampling was employed over random selection because of the study's aim to capture in‐depth, context‐specific perspectives on SRH services utilisation and delivery with direct knowledge and experience of the local SRH landscape, an approach consistent with qualitative research designs, which prioritise information richness over statistical generalisability [20]. A total of 86 participants were interviewed from August until December 2021, only once for the study. Detailed inclusion and exclusion criteria are provided below.

Inclusion criteria for sample selection

A. Inclusion criteria for adolescents to participate in Focus group discussions (FGD) and in‐depth interviews (IDI)

1. Both male and female adolescents aged 15–19 years and residents of four municipalities, namely Bharabise and Chautara municipalities and Balephi and Indrawati rural municipalities.
2. Adolescents (aged 15–17) with their written informed assent and written informed consent from parents/guardians for their participation.
3. Adolescents (aged 18–19) with their written informed consent.

B. Inclusion Criteria for service providers:

1. Health workers such as doctors, health assistants, staff nurses, school health nurses, health facility in‐charges from governmental health facilities and pharmacists from the private sector in the selected municipalities.
2. FCHVs and health coordinators in the selected municipalities.
3. Officials with leadership role at the district health office
4. School teachers teaching health topics from grade 7‐10.

Representatives from community‐based organisations (CSOs) working on adolescent and youth SRHR in the district. Exclusion criteria

1. Adolescents below 15 years of age.
2. Any eligible participants, including adolescents, stakeholders and health workers who declined their consent to participate

### 2.3 Data Collection Tools and Procedures

We used semi‐structured interview guidelines to conduct face‐to‐face FGDs and In‐depth Interviews (IDIs) and Key‐informant Interviews (KIIs). The interview guidelines were adapted from the handbook for conducting an adolescent health service barriers assessment focused on disadvantaged adolescents and contextualised for Nepal [21]. The guide was informed by key constructs of social cognitive theory and organised across individual, social, and environmental domains, including perceived barriers to accessing services, self‐efficacy in addressing SRH challenges, social norms, and observed behaviours influencing service utilisation [22]. Separate but related guidelines were developed for adolescents and key informant stakeholders. The FGD and IDI guide for adolescents included their experiences, perceptions and needs regarding SRH information and services during the COVID‐19 pandemic, including barriers and facilitators to accessing sexuality education and services, including how lockdowns and other COVID‐19 restrictions affected their ability to seek care. The KII guide focuses on their professional experiences and observations of adolescents' SRH service provision, including service availability, accessibility, acceptability and utilisation, their challenges and their responses to make services available during the pandemic. Adolescents and health service providers were consulted during the development and refinement of the interview guidelines to ensure the tools were contextually relevant and responsive to the local SRHR concerns. The guides were piloted among six participants representing each sample group in Sindhupalchowk who were included in this study.

ST, SS (females), and BB (male) conducted the FGDs and IDIs with the support of field researchers, who were public health graduates and were oriented on the objective of the study, qualitative interview skills and research ethics. ST and BB had extensive experience working on adolescent SRHR in Nepal. ST, SS and BB have master's degrees with qualitative research experiences and trainings.

Female FGDs were facilitated by female researchers while male FGDs were moderated by male researchers. Each FGD lasted approximately 120 min while each IDI and KII lasted 90 min which was audio recorded. The interviews were conducted in person in the preferred location of the participants. Field notes were made during the interviews and at the end of the interviews, researchers summarised key points with the participants and reflections from the participants were collected. The interviews were continued until we reached thematic saturation which was discussed and agreed upon within the research team. Transcripts were not returned to the participants for comments or correction.

### 2.4 Ethical Consideration

Ethical approval for this study was obtained from Nepal Health Research Council, the country's national ethical review board (Registration no: 371/2021 P). The study participants received a written ‘Participant Information Sheet’ that included details about the objective of the study, benefits and harm, and confidentiality of their data. Written informed consent was obtained from all participants; for adolescents aged 15–17 years, a written informed assent form was obtained along with written informed consent from their parents or guardians. The audio recordings, transcripts and translated data were stored securely at the BNMT head office and were only accessible to the research team.

### 2.5 Data Processing and Analysis

The recordings were transcribed into verbatim in the Nepali language and then translated into English. All the data were coded in Microsoft Excel using an inductive coding approach. The data identification, analysis and interpretation involved thematic analysis as defined by Braun and Clarke with reading and rereading of the transcripts to get the meaning of the data [23]. The codes with similar characteristics, attribution and meaning were aggregated to form sub‐theme and sub‐themes were aggregated to form themes. ST and BB were engaged in data analysis, review of codebook, development of code tree, and development of the draft codebook. All authors were engaged in reviewing, revising and finalising the code, developing sub‐themes and themes that have been presented as the result (Table 1).

**Table 1:** Participants in In‐depth interviews (IDIs), Focus Group Discussion (FGD) and Key Informant Interviews (KIIs).

S.N	Data collection method	Participant type	No. of FDGs/IDIs/KIIs	Male	Female	Total participants
1.	FGDs	Adolescents	4	26	26	52
2.	IDIs	Adolescents	12	6	6	12
Sub total				**32**	**32**	**64**
3.	KIIs	Female Community Health Volunteers (FCHV's)	4	—	4	4
		School Nurses	2	—	2	2
		School Teacher	2	1	1	2
		Representative of Civil Society Organisation	2	1	1	2
		Health care Providers	4	2	2	4
		Private Practitioners	4	4	—	4
		Health Coordinator	4	4	—	4
Sub total				**12**	**10**	**22**
Grand Total						**86**

*Note:* Bold values are used as a visual formatting preference to distinguish the subtotal and total rows from the individual data rows, to enhance the readibility of the table.

## 3 Results

In total of 149 codes were developed that resulted in the six sub‐themes and six main themes as illustrated in Table 2. The Major themes developed includes:: Theme 1: Pandemic Disruptions Reshaped Adolescents' SRH Information and Care Pathways Theme 2: Constraints within Healthcare System compromises Adolescent SRH Pathways; Theme 3: Realities that Influences Adolescent Sexual Behaviours and Health‐Seeking Practices; Theme 4: Living Through Isolation, Uncertainty, and Educational Disruption; Theme 5: Strengthening self‐efficacy and community agency in emergency preparedness; Theme 6: Social Support enables Adolescent SRH Pathways during Emergencies (Table 3).

**Table 2:** Illustrative examples of the questions used in the guidelines.

IDI/FGD for adolescents	KII for stakeholders
Q3. What do you think are the barriers of young people to getting any sexuality education including SRH information during the pandemic? Do you think the teachers missed or avoided teaching topics of sexuality education of your school curriculum during the pandemic?	Q2. How has COVID‐19 pandemic affected in where and how adolescents get information on SRH and reaching out for help when they need for SRH services?
Q4.1. What are your experiences on the SRH information and services available to you and your peers during the pandemic?	Q2.2 (Accessibility): Do you think the services were available and accessible to the adolescents of your catchment area during the pandemic?
Q4.2. Have you and your peers been able to reach those health services when you needed them during the pandemic? Is it different before the pandemic?	Q2.4. What are your observation and experience of the SRH service utilisation by the adolescent?
Q4.6 What are your thoughts and experience of receiving SRH information and services during the pandemic?	Q2.3 Do you think SRH services of your health facility are adolescent friendly during pandemic?

**Table 3:** Codebook presenting illustrating codes, sub‐themes and themes.

Social cognitive theory (SCT) domain	Themes	Sub themes	Illustrative codes
Environmental	Theme 1: Pandemic Disruptions Reshaped Adolescents' SRH Information and Care Pathways	Subtheme 1: Disruption of school‐based SRH information	no physical class; school health nurse provides counselling: discontinued counselling service from school nurse during lockdown; online classes operated by school; difficulties asking questions online, could not ask questions privately during physical classes; privacy and confidentiality concern during online classes; no good internet facilities at home; increase use of mobile phone; impact peer interaction and relationship; multiple siblings but only one/two phones; overlapping online classes; only one child could attend online classes; do not have smart phone to connect with internet; students left behind in learning; online classes move fast; adolescent could not clearly understand what teacher says; not able to clear the doubt, explanation made by the teachers is not effective in the online class; distraction of taking online classes from home; teacher skips chapters; teacher completes 1 year course in 6 month
		Subtheme 2: Barriers accessing SRH services during lockdown	lack of transportation, shyness accessing services; fear of being judged; concern over privacy; fear of not maintaining confidentiality by health care workers; feeling uncomfortable, girls avoid health facilities due to the presence of male health workers, villagers gossip if girls visit health institutions; feeling shy asking contraceptive, feeling shy asking pad from male health workers; difficulties going to distant health facilities for adolescent due to cost, travel restrictions, police scolding those walking outside; police did not allow them to visit health post; sanitary pad shortages in shop; price hike by the shopkeepers on pad, distance of health facilities, health facilities difficult to reach
		Subtheme 3: Health care adaptation to maintain access to SRH service	service provision during lockdown; knowledge transfers to children; awareness raising activities in the communities, bridge the gap; provide help in need training needs for FCHVs; FCHV requires training first to provide knowledge; training needs for adolescents and teachers; adolescent reaches to FCHV; mobilisation of FCHVs during lockdown; home delivery of condoms and pills; alternative service delivery during pandemic; use of phone appointments; FCHV referring adolescents to health facilities; counselling services by FCHVs; counselling services by health workers over phone; community based awareness; outreach programme; stigma reduction programme; family support
	Theme 2: Constraints within Healthcare System Compromises Adolescent SRH Pathways	Sub Theme 2.1: Health system readiness	Interruption of health post services; health post and health facilities closed during lockdown; family planning campaign halted during COVID‐19; lack of separate room for adolescent in health facilities; shortage of family planning devices; shortage of staff; female health workers not always available; health workers denied family planning services amid COVID surge and priorities; priorities given to COVID response and management; shortage of health manpower, transfer of skilled manpower, health facilities run out of family planning devices
		Sub Theme 2.2: Gaps in health care providers' capacity for adolescent responsive services	lack of separate adolescent‐friendly space; no specific programme targeting to adolescent, health workers doubt about adolescents character when asked for contraceptives; adolescent fear health worker does not maintain confidentiality; health workers keep distance with adolescent when they visit facilities; no counselling by health workers; health care providers refer cases to other health facilities which is often far; fear of being backbitten by community; lack of counselling
Individual	Theme 3: Realities that Influences Adolescent Sexual Behaviours and Health‐Seeking Practices	Sub Theme 3.1: Change in SRH service seeking	increased demand for family planning devices after pandemic; people returned to villages; increase contraceptive use; increased utilisation of health services; reduced facility visits due to fear of COVID infection; shops were open 24 h; used medical shops to buy pads, reached out to FCHVs, counselling and advice, sharing with FCHVs
		Sub Theme 3.2: Changes in sexual behaviour and early pregnancies	Adolescents engage in unsafe sexual activities; unintended pregnancies among adolescents; abortion in adolescents; elopement; early marriage as resolution after sexual relationship; child marriage; friend advice on pregnancy prevention; peer influence on getting married; unsafe sexual intercourse; friends thought getting married during COVID‐19; boy pressuring for marriage; increase in marriage proposals; family problems leading to marriage; school closure leading to marriage; many males are staying home due to job halt or loss
	Theme 4: Living Through Isolation, Uncertainty, and Educational Disruption	Sub Theme 4.1: Social isolation and emotional distress	could not meet friends and talk; adolescents felt stressed and anxious; fear of getting COVID from health facilities; fear of uncertainty; adolescents stayed at home during lockdown; adolescents do not share problems with their own parents; share problems with friends; friends share with adults influenced by friends; increased engagement in social media; less meeting with friends physically
		Sub Theme 4.2: Disrupted education and emerging vulnerability	more free time for adolescents; increase falling in love online; stressful because a course, students may not have understood what I taught, explanation made by the teachers is not as effective in the online class as it was in the physical, distraction of taking online classes, not clearly understood; free time to think, more internet use; play games after getting a mobile phone, mental stress
Social	Theme 5: Strengthening self‐efficacy and community agency in emergency preparedness	Sub Theme 5.1: Skill development for adolescent self‐efficacy	training packages for adolescents; training for adolescent; skill building activities; child club; road play and role play, training on making sanitary pads; self‐made cotton pads
		Sub Theme 5.2: Community actors and volunteers as facilitators of decentralised SRH access for adolescents	Trust in FCHVs; FCHVs provided information; FCHVs providing counselling; school health nurses; come to us without any shyness; hesitation to use health services, feeling shy with male doctors
	Theme 6: Social Support enables Adolescent SRH Pathways during Emergencies	Sub Theme 6.1: Supportive relationship and stigma reduction	SRHR information received from friends and siblings; supportive family; supportive social environment; awareness to parents; knowledge sharing to friends; share problems; negative mind‐set; understanding parents; talk openly with children; confident in seeking services; maintain confidentialities; confidentialities at health facilities
		Sub Theme 6.2: Community awareness and outreach mechanism	awareness raising activities in the communities; awareness to parents; feel supported; outreach programme

A total of 86 participants were included in the study, that comprise 64 adolescents and 22 health care providers and other stakeholders. Of the adolescents, 50% were male and 50% female and all were unmarried and had completed their basic education at the time of the study. The average age of the FGD participants was 16.4 years, representing different castes/ethnicities including: Chhetri 39.1%, Brahmin 12.5%, Janajati 39.1% and Dalit 9.4%. Among health service providers, all school health nurses approached were female, while all health coordinators and private practitioners were male, as no female held these positions in the selected municipalities at the time of data collection, a pattern reflecting broader sex‐aggregation within Nepal's health workforce [24].

### 3.1 Theme 1: Pandemic Disruptions Reshaped Adolescents' SRH Information and Care Pathways

The COVID‐19 pandemic affected adolescents' access to SRH information and services through disruption in education and routine services. Participants describe both barriers and opportunities in accessing SRH information and services during the pandemic.

#### 3.1.1 Disruption of School Based Information

The majority of adolescents shared that they had never accessed SRH services in their lifetime including during the pandemic. Service providers expressed that adolescents' access to SRH information and services was hampered by school closures. A provider shared:

Adolescents remained in their home, if they were in school, they would have an environment to ask. They do not share their [SRH] problems even with their parents. So, if the school was opened then they can get counseling services from the school health nurse like me.

‐ KII, School Health Nurse

Many schools adopted online classes to continue education of adolescents during school closure. However, both adolescents and school health nurses described online classes not being effective to communicate SRHR information to the students. They reported that online classes were not effective in terms of internet connections and the availability of such services to students at their homes.

Similarly, confidentiality and privacy were issues during online classes because it was hard to ask questions or share concerns with teachers while friends were also listening. Further, teachers and students noted that pandemic‐related disruptions led to curriculum compression, limiting time for comprehensive instruction, overlooked or rushed SRHR contents, affecting students' understanding and retention of essential contents.

Compared to physical classes, virtual classes moved too fast. It was difficult to understand the subject, and we could not clarify doubts about SRHR information properly. There were also electricity and internet problems.

‐ FGD, Adolescent Boys

#### 3.1.2 Barriers Accessing SRH Services During Lockdown

Study participants reported that the COVID‐19 pandemic especially during the lockdown period created barriers for accessing SRH services for adolescents. Due to strict lockdown posed by the government authorities to manage and control the COVID‐19 pandemic, adolescents were limited to their homes and could not access the available SRH services at health facilities. Similarly, adolescent girls reported that they encountered difficulties accessing menstrual pads when the shops were closed or difficult to reach. In addition to these physical barriers, social concerns also affected adolescents' willingness to seek SRH services.

Adolescents described feeling shy and uncomfortable discussing SRH‐related issues and concerns about privacy and confidentiality accessing service providers during the pandemic.

During the lockdown, we had to stay at home, making it hard to access services. It was difficult to go out, medical halls were far away, and many places were closed. On top of that, we couldn't openly discuss topics like sexual and reproductive health because of society's negative views on adolescents talking about these issues.

‐ IDI, Adolescent Boy

#### 3.1.3 Health Care Adaptation to Maintain Access to SRH Services

Despite all the challenges and barriers, frontline health workers played a crucial role in ensuring the essential SRH services are available to adolescents. Due to the movement restrictions and limited access to health facilities, female community health volunteers (FCHVs) and health care workers adapted their strategies to meet community needs, including home‐based distribution of family planning commodities and referral support. FCHVs emphasised their accessibility and active role in bridging the gap between communities and health facilities.

During the pandemic, we mobilised female community health volunteers to distribute family planning commodities like condoms and pills. They also gave counselling's and appointments via mobile and called women who preferred depo injections (Depo‐Provera, a birth control shot) from the health facilities.

– KII, Health care provider

Some government and private health care providers reported that, despite the challenges, they continued to offer services to adolescents and the general population during the lockdown period.

The health facility was closed for 10‐11 days because many staff members tested positive for COVID. However, other staff who tested negative were still available to provide services. Apart from the days it was sealed, the health facility's services were quite effective.

‐ KII, Health care provider

### 3.2 Theme 2: Constraints Within Healthcare System Compromise Adolescent SRH Pathways

The second theme identified was health system barriers during the pandemic. Participants described challenges related to the readiness of the system and gaps in health care providers' capacity to address SRH needs of the adolescents.

#### 3.2.1 Health System Readiness

Some health care providers reported that there were shortages of supplies and even human resources during the lockdown period of COIVD‐19.

During the pandemic, we faced shortages of family planning devices, including pills and Depo‐Provera (Depo contraceptive injections). There was a nationwide shortage for a time, but now we have all the supplies we need as usual

‐ KII, Health Office

Despite the challenges and obstacles, different study participants highlighted the areas that could increase resilience for SRH services among adolescents during the pandemic. Health care providers reported that clear coordination and defined authority across different levels of the government are required for effective SRH service delivery for adolescents during crises. They emphasised the role of local municipalities in ensuring functional, responsive and uninterrupted SRH services, while higher provincial and central government should play coordination or facilitation roles.

We have federal, provincial, and local governments, but coordination is weak, which leads to duplication and inefficiencies. At the local level, human resources are limited, and programmes often deviate from their intended norms. We do not necessarily have the authority to solve many issues; our role is mainly to coordinate and facilitate. During crises, having clearer roles and decision‐making authority at one level could make implementation much more effective.

‐ KII, Health Coordinator

Along with coordination among government system, participants highlighted the importance of strengthening health service delivery through ensuring trained staff, maintaining privacy in the health facilities, and continuous availability of medical supplies. Local municipalities were considered key actors in guaranteeing these facilities.

Local governments need to take proactive ownership of adolescent SRHR. They should understand how many adolescents live in their area, what challenges they face especially in times of crisis like the pandemic and organise community discussions around these needs. Local governments must also ensure rules and regulations to mobilise human resources effectively and allocate a separate budget for adolescent SRHR, so that services remain accessible and effective during emergencies

‐ KII, FCHV

A key informant in this study highlighted the need for collaboration between local government and non‐governmental organisations for effective management of adolescent SRHR issues during the pandemic.

NGOs play a key role in addressing sexual and reproductive health issues in society. We act as a bridge between the community and government, bringing local problems to national attention and helping to create action plans. Collaboration with local and provincial governments is essential, especially to focus more on adolescent education and awareness.

‐ KII, CSO

#### 3.2.2 Gaps in Health Care Provider's Capacity for Adolescent Responsive Services

Government health care providers reported that their priorities shifted to managing and responding to COVID‐19 which impacted the delivery of adolescent‐focused services from the hospital and health facilities, especially primary health centres of Sindhupalchowk. Health coordinators of two different municipalities reported that the adolescents' health needs and issues were not prioritised during the pandemic.

During the COVID‐19 period, we only provided services through a fever clinic.” As a result, there were significant obstacles to other services. Except for the fever clinic and some emergency cases, all other services were greatly reduced, making it seem like other services were almost completely closed. We had a shortage of health staff because some trained workers were moved to different roles, and there weren't enough people to cover all the needs.

‐ KII, Health Coordinator

The health care providers also highlighted that the SRH information and services for adolescents and health facilities providing such services were also overshadowed by the COVID‐19 response and management priority. The health care providers acknowledged the need for training to respond to SRH needs of adolescents during emergencies such as COVID‐19. Similarly, the adolescent‐friendly sites were also used to screen and treat COVID‐19 patients.

We were primarily focused on COVID‐19 management, such as swab collection and vaccination. While we continued with regular programmes, we did not have any special initiatives for adolescents. In this situation, our attention shifted to maternal and child health, and unfortunately, services related to adolescents were overlooked, which could have been prioritized.

‐ KII, Health Coordinator

### 3.3 Theme 3: Realities That Influences Adolescent Sexual Behaviours and Health‐Seeking Practices

The third theme concerned perception of adolescent behaviour during the COVID‐19 pandemic. Participants described changes in adolescents' SRH service‐seeking practices and sexual behaviour during the pandemic, including perceived changes in sexual activities and early pregnancies.

#### 3.3.1 Change in SRH Service Seeking

Health care providers described changes in adolescents' health‐seeking patterns during COVID‐19. Providers from the government health facilities reported that more adolescent girls visited their health facilities for SRH services during the lockdown, particularly as private health facilities and pharmacies were closed. They also reported an increase in contraceptive demand during the pandemic.

Before the pandemic, boys came to us for condoms, which were normal. However, during the pandemic, adolescent girls started coming to us for help because private medical shops were closed.

‐ KII, Health care provider

During the pandemic, adolescents sought services from the health workers when experiencing problems. Adolescents especially reached to FCHVs for family planning devices, consultation and counselling for SRH.

There was one adolescent girl who shared her concern over the phone that she had not had her period for two months. I advised her to have a urine pregnancy test at the health facility. Later, she developed a fever and went to the health facility. At that time, I reminded her to have a pregnancy test first before taking any medicines. She is now pregnant and is currently 19 years old.

‐ KII, FCHV

#### 3.3.2 Changes in Sexual Behaviour and Early Pregnancies

Adolescents perceived that COVID‐19 impacted the rise in early marriage and pregnancy among adolescents.

I think marriage rates increased during the lockdown. Many of my friends got married during this time. One even eloped because her parents took away her mobile phone. Many unmarried friends had unsafe sex and got pregnant”

‐ IDI, Adolescent girl

Adolescent girls also reported an increase in sexual activities during the pandemic period, attributing this rise to the additional leisure time they had.

My classmate's sister, who is younger and studied in class 8, was in a relationship with a boy who was older than her and studying in 10 + 2. She had unsafe sexual intercourse with him and unfortunately got pregnant. Her sister and I went to health post and told the health workers about the incident.

‐ FGD, Adolescent girls

Some health workers, however, contend that early marriage was already prevalent in the community before the pandemic and that the pandemic itself did not contribute to this trend.

The health care providers perceived that the use of social media was a cause of early marriage. The participants of the study stated that family problems, pressure from boys and families, no school engagement and high access to and use of mobile phones and internet contributed to child marriage. One participant noted that sexual activities and marriages may have actually decreased due to movement restrictions and the difficulty of leaving home while schools were closed.

Child marriage was already prevalent in the community prior to the pandemic. However, increased access to mobile phones and the internet during the pandemic has contributed to a rise in the cases. The lockdown exacerbated problems for adolescents, as many shops were closed, making it difficult to obtain family planning devices. Although government health services remained open, people initially avoided seeking help due to familiarity with local health service providers. As the lockdown extended and other options became limited, more individuals eventually reached out to address their SRH issues.

‐ KII, Health care provider

### 3.4 Theme 4: Living Through Isolation, Uncertainty, and Educational Disruption

The fourth theme concerned the psychosocial and mental health effects of the COVID‐19 pandemic on adolescents. Participants described feelings of loneliness and emotional distress associated with fear, loneliness and disruption of their daily life and education. Participants also perceived that these disruptions increased adolescents' vulnerability to potential harmful behaviour.

#### 3.4.1 Social Isolation and Emotional Distress

Both participants in this study reported compromised psychosocial and mental well‐being, increased feelings of isolation, anxiety, and risky coping behaviours. Due to the closure of schools and reduced peer interaction, adolescents reported a sense of loneliness and emotional distress.

When I was at school, I could meet and talk with my friends, but due to this pandemic, the school was closed, I could not go anywhere because of the fear of transmission. It was a difficult time. We felt isolated.

‐ FGD Girls

Health care providers also acknowledged the increased levels of stress and anxiety among adolescents during pandemic; however, these feelings were not only due to the fear of COVID‐19 but also due to the lack of access to SRHR information and also unmet needs for sexual health.

During this period (COVID‐19 pandemic) mental health was affected along with anxiety problems. Especially most of the adolescents did not even know about COVID‐19 and the same thing implies to their sexual and reproductive health and rights. There was a fear among people on visiting a health facility for receiving sexual and reproductive health services.

‐ KII, Health care provider (Private)

#### 3.4.2 Disrupted Education and Emerging Vulnerability

The pandemic aggravated emotional stress and loneliness among adolescents, and it is perceived that such psychosocial distress may lead to harmful behaviours. The loss of school and work routine created opportunities for adolescents to spend time communicating with intimate partners, which the participants perceived as contributing to early marriage or living together.

There was neither study nor work. When they stayed at home, they had a lot of free time, which may have influenced their thoughts and behaviour. Because of this, they want to get married or live together. So many of my brothers and sisters got married this way due to studies being stopped and works being stopped for those who were also involved in work.

Adolescent girl, Barbarise

#### 3.4.3 One Health Coordinator States

The adolescents have not been able to continue their education in person, and they might have been suffering from stress and anxiety. This might also contribute to some psychosocial problems in them. Adolescents could be engaged in drug abuse or sexual activities.

‐ KII, Health coordinator

Disruption to education during the pandemic was also contributing to increased academic pressure and potential learning gaps among adolescents. One teacher described about the challenges to complete 1 year course within 6 months, as a result the teacher perceived that it had created pressure on students' learning and potentially contributed to psychosocial distress.

It was stressful because a course that was supposed to be completed in one year had to be completed in six months. I had to teach very quickly and skip some chapters. I think some students may not have understood what I taught; this is my personal opinion.

–KII, School teacher

### 3.5 Theme 5: Strengthening Self‐Efficacy and Community Agency in Emergency Preparedness

The fifth theme discussed factors that support adolescent self‐efficacy and community agencies in maintaining access to SRH information and services during the pandemic. Participants highlighted the importance of developing adolescents' skills and knowledge, as well as the role of community‐based actors in facilitating access to SRH support.

#### 3.5.1 Skill Development for Adolescent Self‐Efficacy

Health care providers and adolescents highlighted the need of improving adolescent skills and knowledge to ensure resilience during crises such as COVID‐ 9 pandemic. Both emphasised on the need of trainings and active engagement of adolescents through child clubs and practical skill and knowledge development initiatives to both adolescents and FCHVs could enable access to essential resources and information during crisis situation. Child clubs are community‐based, child‐led groups formed in Nepal since the late 1990s to promote children's participation in decision‐making, awareness raising, peer support and skills development in line with Nepal's commitment to the UN Convention on the Rights of the child [25].

Our school child club exists only in name. If the children and teachers were trained and actively involved, they could work for themselves and even support other students. I also need proper training first so I can teach them effectively. If everyone had knowledge and guidance, the child club could play an important role in supporting adolescents, especially during difficult times like the pandemic.

‐ KII, Health care provider

To address the shortage of SRH commodities, some local government and community organisation conducted training to produce SRH commodities during pandemic; an adolescent girl shares her insights:

We did not face problem with sanitary pads because our school taught us how to make cloth pads. So, during shortage of sanitary pads, we used those self‐made home pad instead. Since the market was also nearby, we could buy pads when needed. But for those living farther away, it might have been difficult. If they had also received the same training, they could have managed better during pad shortages.

‐ IDI, Adolescent girl

#### 3.5.2 Community Actors and Volunteers as Facilitators of Decentralised SRH Access for Adolescent

Both service providers and adolescents emphasised on the beneficial role of FCHV's in their communities and highlighted the need of strengthening and mobilising FCHVs in the communities. Participants described FCHVs as trusted frontline workers who bridge the gap between communities and health facilities. They noted that empowering FCHVs with training, resources, and recognition is key to ensuring adolescents, especially in rural areas and have access to timely SRH information and support.

Many adolescents still hesitate to trust them, fearing their problems might spread within the community, therefore, we provided training to FCHVs to assure communities that health facilities maintain full confidentiality in cases like unwanted pregnancy, or other sexual or reproductive health problems to adolescents.

KII, Health care provider

Both adolescent girls and boys preferred same gender service providers to ask questions and seek information regarding SRH services, which was missing in the study areas. Participants reflected on the gender socialisation process and its influence in seeking SRH information and services, suggesting the role of key community actors.

If the service providers were males at health posts, I would personally go there for information and services on sexual and reproductive health. When I was younger, I used to take condoms from health posts, but as I grew up, many recognized me and I felt shy to go there. If anyone saw me taking condoms, people would think I am ‘bad,’ and this information would spread quickly in the community.

‐ IDI, Adolescent boy

### 3.6 Theme 6. Social Support Enables Adolescent SRH Pathways During Emergencies

The sixth theme highlights the importance of social support and community engagement in creating a supportive environment for adolescents to access SRHR information and service pathways during the pandemic. Participants emphasised the role of supportive relationships and stigma reduction as well as community awareness and outreach activities, in facilitating adolescents' access to SRH support.

#### 3.6.1 Supportive Relationship and Stigma Reduction

Adolescents emphasised on the importance of a supportive family and social environment to reduce the stigma around adolescents' SRHR. Awareness to parents and communities and maintaining confidentiality at health facilities is crucial to increase health services by the adolescents.

It really depends on the family environment. In families where these topics are discussed and parents are understanding, adolescents feel comfortable sharing their problems. But in families where such conversations don't happen, adolescents tend to feel shy and usually don't share their concerns. To make things better, parents should be encouraged to talk openly with their children about sexual and reproductive health, so that adolescents feel supported and confident in seeking the services they need.

‐ FGD, Adolescent boys

#### 3.6.2 Community Awareness and Outreach Mechanism

Health care providers emphasised the importance of community‐based awareness and outreach programme to improve resilience for SRH services for adolescents during the pandemic. They suggested that role play, street dramas, radio and television could be effective in raising awareness alongside adequate staffing and resources.

If government really wants to reduce barriers for adolescents, it should start with awareness raising programme using radio, TV, even community dramas to reach people. We have adolescent‐friendly health facilities, but they are not effective because trained staffs are lacking and not replaced when transferred. The government should ensure these posts are filled and provide family planning devices that meet adolescents' needs. “Only then can the services truly become adolescent‐friendly.

‐ KII, Health care provider

## 4 Discussion

This study explores how the COVID‐19 pandemic affected adolescents' access to and utilisation of SRH information and services in Nepal and identified the barriers and facilitators influencing their SRH information and service pathways. Viewed through a social cognitive framework, the findings of this study illustrate how individual, social and environmental factors interact to shape adolescents' SRH information and service pathways during the pandemic, which is unique.

The study highlighted that the pandemic limited adolescents' access to their SRH knowledge and information mainly due to environmental factors such as school closure, inequality in digital equipment, and limited media literacy among adolescents. When schools closed, adolescents lost structured and trusted sources of information, potentially increasing their reliance on informal or less reliable sources. Previous studies compliments these findings and report gaps in adolescents' access to SRH information during emergencies, including greater reliance on informal sources and inequalities in access to alternative sources of information [9, 26]. Extending to those studies, findings from this study further highlight lost opportunities to interact with teachers and peers, ask questions, clarify doubts and learn through these social interactions. Such disruption of in‐person educational environments and reduced interaction with trusted sources such as teachers and peers may have limited adolescents' opportunities for social learning and made it more difficult for them to access and understand SRH information. Some of the existing studies suggest that peer relationships and socialisation contribute to adolescent wellbeing [27, 28, 29]. For some adolescents, online classes did not fully replace these opportunities because of limited access to digital devices, shared phones, poor internet connectivity and difficulties understanding or asking questions during online classes. This study recommends continuity plans for adolescents' SRH education including alternative digital or community approaches must be developed during emergencies.

Adolescents faced challenges related to confidentiality, provider attitudes, availability of female providers, counselling and adolescent‐friendly services. Thus, even when some services continued, adolescents may not have experienced them as accessible or acceptable. These system‐level constraints may have affected adolescents' ability and willingness to seek services. The findings suggest that facility‐based adolescent SRH services are particularly vulnerable during health emergencies and should therefore be explicitly incorporated into emergency preparedness and service continuity plans. This finding is similar to other evidence from Nepal and other low‐resource settings, which showed limited SRH service utilisation in general and during pandemic interacted with existing social, cultural and gender barriers to access SRH services [14, 30]. Adolescents also reported changes in sexual and reproductive behaviours, alongside anxiety, stress and social isolation. The findings of this study uniquely highlight supportive family relationships and active engagement of community health workers (such as FCHVs).

Some of the key individual behavioural findings that this study stress is the perceived increase in early marriages, unintended pregnancies, and sexual activities during the COVID‐19 pandemic. These findings are consistent with evidence from other settings showing that public health emergencies can contribute to early marriage, unintended pregnancies and changes in sexual behaviour [31, 32, 33]. However, our findings provide further insights into the circumstances surrounding these reported behavioural changes. Participants described school closures, reduced social interaction, peer influence and reduced access to SRH information and services as part of the context which these behaviours occurred. The findings are consistent with prior research demonstrating that public health emergencies disproportionately affect adolescent health behaviours and access to services [31, 34, 35]. Such disruption of SRH services may also have affected adolescents' confidence and willingness to seek care, particularly where they experienced uncertainty about the service availability, confidentiality or provider support [36, 37, 38].

Our findings show that adolescents did not respond to these disruptions in the same way. Some participants described reduced visits to health facilities because of fear of COVID‐19 or difficulties reaching services, while others reported increased contraceptive use or sought information and support through friends, pharmacies and FCHVs. This variation is important because it shows that disruption did not result in a single pattern of health‐seeking behaviour, instead, adolescents adapted their pathways according to the support, information and services available to them. This suggests that adolescents adapted their SRH information and service‐seeking practices in different ways as formal services and social environment changed. Reduced access to contraception, SRH information, and confidential adolescent responsive services may have increased adolescents' vulnerability to unintended SRH outcomes, while alternative sources of support may have helped some adolescents navigate these disruptions.

Another key insight of this study was the adolescents' experience of anxiety and stress during the pandemic, mainly due to school closures and reduced social interaction with peers and community support systems. Similar findings have been reported in other settings where pandemic‐related uncertainty and restrictions caused emotional distress among adolescents and young people [39, 40, 41]. Our findings further show that this distress was closely linked to the disruption of adolescents' everyday social relationships. Participants described being unable to meet or talk with friends, spending more time at home and having difficulty sharing their concerns with parents, while some turned to friends and social media for support. This suggests that the loss of social interaction during the pandemic affected not only adolescents' emotional wellbeing but also the way in which they accessed support and discussed sensitive concerns.

These individual‐level factors were closely connected with the social and environmental changes created by the pandemic and may have affected adolescents' confidence and ability to seek SRH information and services may have influenced SRH‐related behaviour and reduced their ability to seek and utilise appropriate services. Therefore, the findings suggest that adolescents' SRH needs during public health emergencies extended beyond the availability of clinical services and include opportunities for emotional, social and trusted support. Integrating mental health and psychosocial support into adolescent responsive SHR services may therefore be important for maintaining adolescents' wellbeing and access to care during emergencies.

Social factors played a critical role in shaping adolescents' SRH outcomes during the pandemic. Our findings further demonstrate that social support was not simply an additional source of information; it could also help adolescents gain confidence to discuss sensitive SRH concerns, seek advice and reach appropriate sources of services. One of the key insights from this study is the importance of orientation and training on SRH for adolescents to enhance their confidence to seek information and services. The involvement of parents, teachers, peers, FCHV's and other community actors therefore provided different points of support when usual school‐ and health facility‐based pathways were distrusted in emergencies. Sensitisation sessions for parents and teachers to initiate SRH discussion at home and school, community awareness‐raising initiatives, mobilising community actors, volunteers and champions may help decentralise access to SRH information and services and support positive norms and behaviour during emergencies. Taken together, these findings suggest that community and interpersonal support can provide important alternative pathways when formal systems are disrupted, while also strengthening adolescents' confidence and capacity to seek appropriate care. Effective adolescent health programmes need to intervene at multiple levels ranging from individual, family, peer and community to institutional and policy levels.

The multi‐sectoral challenges for adolescents' SRH service continuity and utilisation during the pandemic highlight the need to strengthen resilience across the health system and the communities in which adolescents live and seek support. An important contribution of this study is that resilience extends beyond the continuation of SRH services and stresses adolescent agency and support system. These findings on health system and community resilience are consistent with the existing studies on health system resilience to sustain positive individual and community behaviour, promoting self‐efficacy and enhancing local capacities to address the challenges imposed by pandemic in continuity of service [42, 43, 44]. Thus, it will be essential to strengthen SRH services for adolescents by mobilising child clubs through virtual networks as a self‐help group where adolescents can discuss the SRH need, access to SRH information and services, while maintaining appropriate links with trusted adults and health providers.

Furthermore, it would be important to train health care providers to ensure confidentiality and non‐judgmental care for adolescents accessing SRH services in humanitarian contexts including pandemic. Multiple other studies also suggest integrating training programmes such as minimum initial service package (MISP), mental health counselling, and orientation on referral services to health care providers for quality service provision during humanitarian contexts such as pandemics and climate‐induced disasters [45, 46]. Furthermore, the study findings highlight the limited capacity of the health system and health care providers' ability to respond to the pandemic and prioritise SRH services during the pandemic. Service providers reported that their efforts were redirected towards pandemic response while deprioritising adolescent health during pandemic and health facilities struggled to sustain services that have provision of adolescent‐friendly services. These findings extend previous evidence by showing that service disruption was not limited to whether a facility remained open or whether a service was technically available. Furthermore, during emergency situations, psychosocial support could be facilitated by the school health nurse and health care providers from the community leveraging existing digital innovation.

### 4.1 Strength and Limitation of the Study

The study has several strengths. First, it has included both adolescents and key informants, including health care providers, which helped triangulation of the data and captured complementary perspectives on adolescents' experiences of SRH information and services access and the challenges faced by providers during pandemic. The study also examined adolescents' SRH from a multidimensional perspective, encompassing access to information and services, health system barriers, behavioural changes, psychosocial effects and social and community support. The study also identified community‐based adaptations and facilitators that may inform strategies to maintain adolescent‐responsive SRH services during future public health emergencies. The study tool was guided by social cognitive theory, which emphasises an ecosystem approach in understanding personal, social, and environmental influences on access to and use of SRH information and service pathways among adolescents during pandemic like COVID‐19. Another strength of this study was the stakeholder‐informed development and refinement of methodology and study tools, which grounded the research in context‐specific insights and provided rich data.

Apart from the above‐mentioned strengths, this study has few limitations. First, the study was conducted only in a single district of Nepal, providing context‐specific information. Further, Sindhupalchowk was among the highly affected district by the 2015 earthquake that resulted in disrupted SRH services. The verbatim from health workers about increased child marriages even before the pandemic reflects this limitation. Thus, careful considerations should be made while generalising it to other districts of Nepal. We suggest using similar multiple studies that represent different contexts in Nepal to understand perspectives and experiences of service users and providers in developing local solutions for continuation and optimal utilisation of SRH services by adolescents during the pandemic. Second, this study is prone to social desirability bias as SRH issues are highly stigmatised in communities in Nepal, including Sindhupalchowk district. This is reflected in the study findings where majority of the adolescent respondents reported never accessing SRH services during and before the pandemic.

## 5 Conclusion

The COVID‐19 pandemic disrupted adolescent access to SRH information and services, with particular implications for girls. The findings demonstrate that these disruptions were not limited to the availability of health services but occurred through changes in the wider environment, social relationships and individual experiences. School closures disrupted access to school‐based SRH information and opportunities to interact with teachers and peers, while health system constraints affected the availability and responsiveness of SRH services. At the same time, adolescents experienced changes in sexual and reproductive behaviours, social isolation, anxiety and stress, as well as concerns related to stigma and confidentialities. Together, these factors disrupted the ways in which adolescents obtained information, sought support and accessed SRH services during the pandemic.

The finding also identifies barriers and source of support that influenced adolescents' SRH information and service pathways during the pandemic. Health system and service constraints, limited access to information, stigma, confidentiality concerns and reduced social interaction created barriers to SRH service use. Similarly, maintaining adolescents' SRH care pathways during public health emergencies requires an ecosystem approach that addresses individual, social and environmental factors together. Supportive families and peers, FCHVs, strengthening community‐based support, adolescent responsive and confidential services, strengthening the capacity of health care providers and community actors as part of emergency preparedness and service continuity planning are crucial to help adolescents navigate disruption in SRH information and care, ultimately supporting their health and wellbeing during emergencies.

## Author Contributions

**Saki Thapa:** conceptualisation, methodology, software, data curation, investigation, validation, formal analysis, supervision, funding acquisition, visualisation, project administration, resources, writing – original draft, writing – review and editing. **Bikram Bucha:** conceptualisation, methodology, software, data curation, investigation, validation, formal analysis, project administration, resources, writing – review and editing. **Swastika Shrestha:** conceptualisation, data curation, investigation, validation, writing – review and editing. **Raghu Dhital:** funding acquisition, supervision, writing – review and editing. **Kritika Dixit:** validation, writing – review and editing. **Lal Rawal:** supervision, validation, writing – review and editing. **Amit Timilsina:** methodology, software, supervision, validation, visualisation, writing – review and editing.

## Ethics Statement

Ethical approval was obtained from Nepal Health Research Council (Ethical Review Board, Registration no: 371/2021 P on 29th July, 2021). All ethical aspect of research was fully adhered during data collection, analysis, and manuscript development.

## Consent

Written informed consent was obtained from all participants prior to participation in the study. For adolescent participants, consent was additionally obtained from their parents or legal guardians, in accordance with ethical guidelines. Participants were informed about the purpose of the study, confidentiality measures, and their right to withdraw at any time without consequence.

## Conflicts of Interest

The authors declare no conflicts of interest.

## Data Availability Statement

The dataset generated and analysed during the current study are not publicly available due to ethical and confidential consideration involving adolescent participants. Relevant illustrative excerpts are presented in the manuscript. Further information may be obtained from the corresponding author upon reasonable request.
